# *TP53* minigene analysis of 161 sequence changes provides evidence for role of spatial constraint and regulatory elements on variant-induced splicing impact

**DOI:** 10.1038/s41525-025-00498-0

**Published:** 2025-05-08

**Authors:** Daffodil M. Canson, Inés Llinares-Burguet, Cristina Fortuno, Lara Sanoguera-Miralles, Elena Bueno-Martínez, Miguel de la Hoya, Amanda B. Spurdle, Eladio A. Velasco-Sampedro

**Affiliations:** 1 Population Health Program, QIMR Berghofer, Herston, QLD 4006 Australia; 2 Splicing and genetic susceptibility to cancer. Instituto de Biomedicina y Genética Molecular de Valladolid (IBGM). Consejo Superior de Investigaciones Científicas - Universidad de Valladolid (CSIC-UVa), 47003 Valladolid, Spain; 3 Molecular Oncology Laboratory CIBERONC, Hospital Clínico San Carlos, IdISSC (Instituto de Investigación Sanitaria del Hospital Clínico San Carlos), Madrid, Spain; 4 Faculty of Medicine, The University of Queensland, Herston, QLD 4006 Australia

**Keywords:** Cancer genetics, Molecular medicine

## Abstract

We investigated the role of *TP53* splicing regulatory elements (SREs) using exons 3 and 6 and their downstream introns as models. Minigene microdeletion assays revealed four SRE-rich intervals: c.573_598, c.618_641, c.653_669 and c.672+14_672 + 36. A diagnostically reported deletion c.655_670del, overlapping an SRE-rich interval, induced an in-frame transcript Δ(E6q21) from new donor site usage. Deletion of at least four intron 6 G-runs led to 100% aberrant transcript expression. Additionally, assay results suggested a donor-to-branchpoint distance <50 nt for complete splicing aberration due to spatial constraint, and >75 nt for low risk of splicing abnormality. Overall, splicing data for 134 single nucleotide variants (SNVs) and 27 deletions in *TP53* demonstrated that SRE-disrupting SNVs have weak splicing impact (up to 26% exon skipping), while deletions spanning multiple SREs have profound splicing effects. Our findings may prove relevant for identifying novel germline *TP53* variants causing hereditary cancer predisposition and/or somatic variants contributing to tumorigenesis.

## Introduction

Pre-mRNA splicing, the removal of introns followed by exon ligation to produce the mature mRNA, is a key step in the expression of most human genes. Alternative splicing plays a vital role in controlling gene expression and enhances the complexity of the transcriptome and proteome by allowing a single gene to produce multiple mRNA transcripts^1^. Constitutive and alternative pre-mRNA splicing are controlled by a wide array of factors and sequence motifs including the 5’ (donor) and the 3’ (acceptor) splice sites, the polypyrimidine tract (PPT), the branch point (BP), and splicing regulatory elements (SREs) such as exonic or intronic splicing enhancers (ESE/ISE) or exonic or intronic splicing silencers (ESS/ISS)^2^. *Trans*-acting RNA binding proteins (RBPs) known as splicing factors regulate exon inclusion, exclusion, or alternative splice site usage by binding to SREs within the pre-mRNA exons or their flanking introns^3^. The commonly recognized RBPs that target SREs include, but are not limited to, serine/arginine-rich (SR) proteins and heterogeneous nuclear ribonucleoproteins (hnRNPs)^4^. Splicing factors and SREs regulate splicing in a position- and context-dependent manner^4^. For example, RBPs of the hnRNP F/H gene family bind to sequences of three or more consecutive guanines (G-runs) that act as enhancers when located downstream of a donor site^5^, and conversely as silencers when located in the exon^6^. Genetic variants that affect splicing motifs and disrupt the binding of splicing factors can cause defective splicing resulting in abnormal mRNA transcripts, or modify the relative levels of alternatively spliced isoforms. Splicing alterations are causally associated with cancer susceptibility, development, progression, and prognosis^7,8^.

The *TP53* gene is composed of 11 exons and two cryptic exons (9β and 9γ) within intron 9^9^. *TP53* alternative splicing, alternative promoter usage, and alternative initiation of translation generates at least 16 different p53 protein isoforms^10^. Several studies have shown that p53 isoforms are abnormally expressed in a wide array of cancers^9^. For example, elevated expression of the Δ40p53 isoform is associated with the aggressive triple negative breast cancer^11^. *TP53* intronic variants can affect the production of alternative p53 isoforms. Minigene experiments measuring the effect of two G-run deletions and site-directed mutagenesis of these G-runs in intron 3 showed increased levels of mRNA with intron 2 retention that encodes the Δ40p53 isoform^12^. Somatic variants at the intron 4 donor site are associated with overexpression of transcripts encoding the Δ133p53 isoforms in breast tumors^13^. Somatic variants can also produce aberrant isoforms such as the intron 6 acceptor site variants that activate an intronic cryptic acceptor, generating the truncated p53Ψ isoform that promotes metastasis^14^. Moreover, *TP53* rare germline variants that cause splicing aberrations are associated with Li Fraumeni Syndrome^15,16^, a hereditary multicancer predisposition syndrome characterized by early-onset cancers primarily breast cancer, bone and soft tissue sarcomas, brain tumors, and adrenocortical carcinomas^17^.

Previous studies on variant-induced *TP53* aberrant or alternative splicing have mostly focused on the donor and acceptor splice consensus motifs^18–20^. We previously assessed presumed missense and synonymous variants in *TP53* for splicing impact through abrogation or creation of donor/acceptor splice site motifs or activation of cryptic splice sites^21^. In this study, we investigated the splicing impact of variants located in *TP53* SRE-rich regions. We prioritized *TP53* exons 3 and 6, as we consider both exons good candidates for SRE-dependent exon recognition, albeit for different reasons. First, the *TP53* exon 3 size (22 nt) falls into the category of “microexons” ( < 30 or 51-nt depending on the authors), which would require specialized regulatory factors involved in their recognition and inclusion^22,23^. Second, the weakness of exon 6 native donor site, as well as the presence of a strong intronic cryptic donor site 63 nt downstream, suggest a specific regulation that would promote recognition of the native donor site through SREs. Further, *TP53* introns 3 and 6 contain several G-rich sequences that represent putative binding motifs for splicing factors, such as hnRNP A/B and F/H that play a role in intron definition^24^. Notably, intron 3 contains G-quadruplex structures that control intron 2 excision in H1299 cells^12^.

In previous reports, we have shown that minigene analysis is a suitable approach for studying the splicing effect of variants in cancer susceptibility genes^21,25–29^. The experimental data generated from these previous minigene assays had been used in clinical variant classification. In our recent study^21^, we designed a splicing reporter minigene with *TP53* exons 2–9 (mgTP53_2-9), where we tested missense and synonymous variants, then applied the splicing assay data to classify the variants according to the ClinGen Sequence Variant Interpretation Splicing Subgroup recommendations^30^. Here, we used the minigene mgTP53_2-9 to test for the presence of SRE-rich intervals by analyzing 23 different microdeletions in exons 3 and 6 and their downstream introns. We also tested the impact of four deletions detected in patients (reported in the ClinVar database) and 134 single nucleotide variants (SNVs) on *TP53* splicing. We aimed to answer two key questions that would contribute to improved classification of variants outside the splice consensus motifs: (1) To what extent do *TP53* SRE-disrupting variants alter the native splicing patterns of this gene? (2) Can splicing impact of these variants be accurately predicted using current computational tools? In addition, we conducted a secondary analysis to predict the RBPs potentially targeting the *cis*-regulatory elements, thereby illuminating potential molecular mechanisms and suggesting specific avenues for future investigation.

## Results

**Fig. 1:**
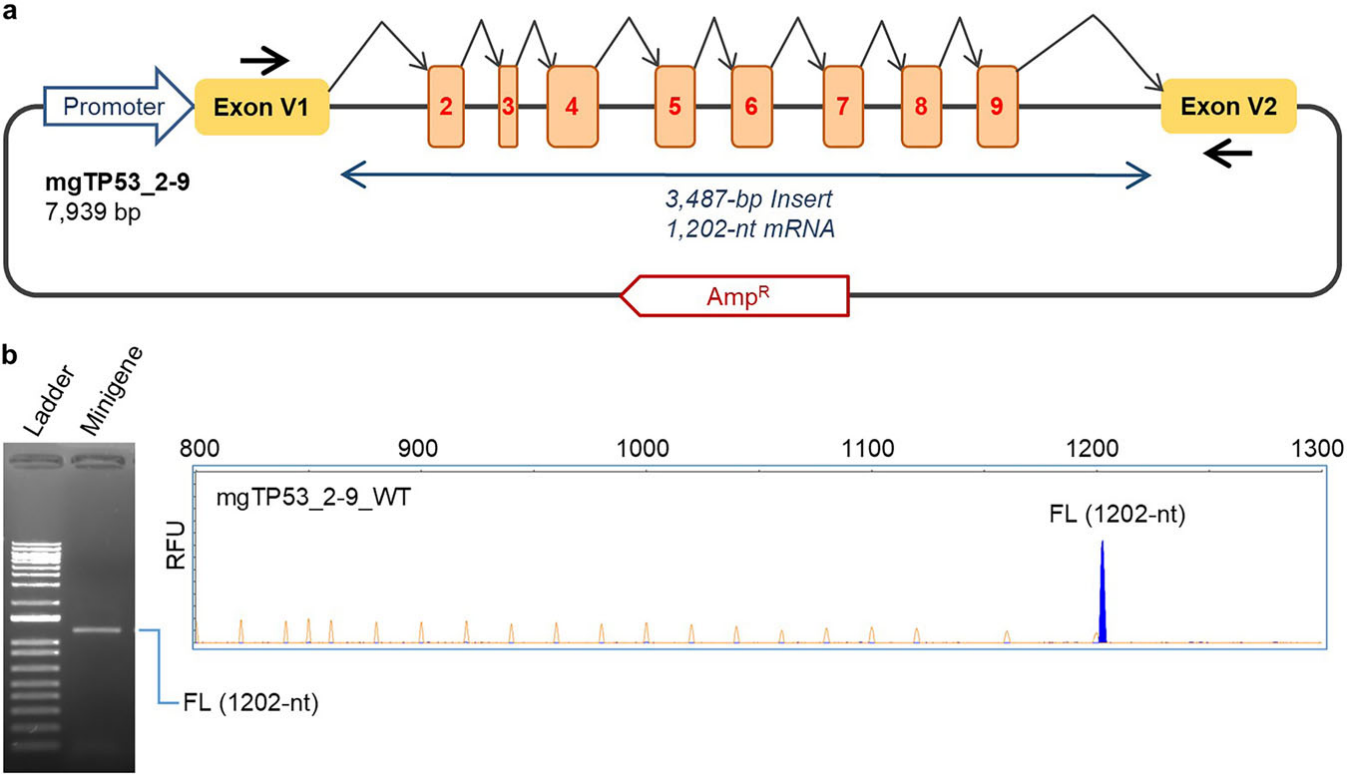
Structure and splicing assay of the *TP53* minigene with exons 2 to 9. **a** Schematic representation of the minigene mgTP53_2–9 with exons 2–9 in numbered boxes. Black arrows indicate the location of vector-specific RT-PCR primers. **b** Splicing assay of the WT minigene. RT-PCR products were analyzed by agarose gel electrophoresis (left) and fluorescent fragment electrophoresis (right). FL, expected minigene full-length transcript. The x-axis indicates size in bp and the y-axis represents Relative Fluorescence Units (RFU).

The minigene construct mgTP53_2-9 (Fig. 1a) produced the expected FL transcript of 1202-nt (V1 – *TP53* exons 2 to 9 – V2) without any alternative transcript when assayed in SKBR3 cells (Fig. 1b), resembling the naturally occurring pattern found in breast and ovary (data not shown). Additionally, the splicing patterns produced from SKBR3, U2OS, and HeLa cells were identical on agarose gel, indicating reproducibility of splicing profile (Supplementary Fig. S1). The SpliceVault^31^ 300K-RNA data and its sub-group of breast samples also showed a low frequency of alternative splicing events in *TP53* exons 2 to 9, supporting a very low level of naturally occurring alternative splicing in this region.

### Functional mapping of splicing regulatory elements

**Fig. 2:**
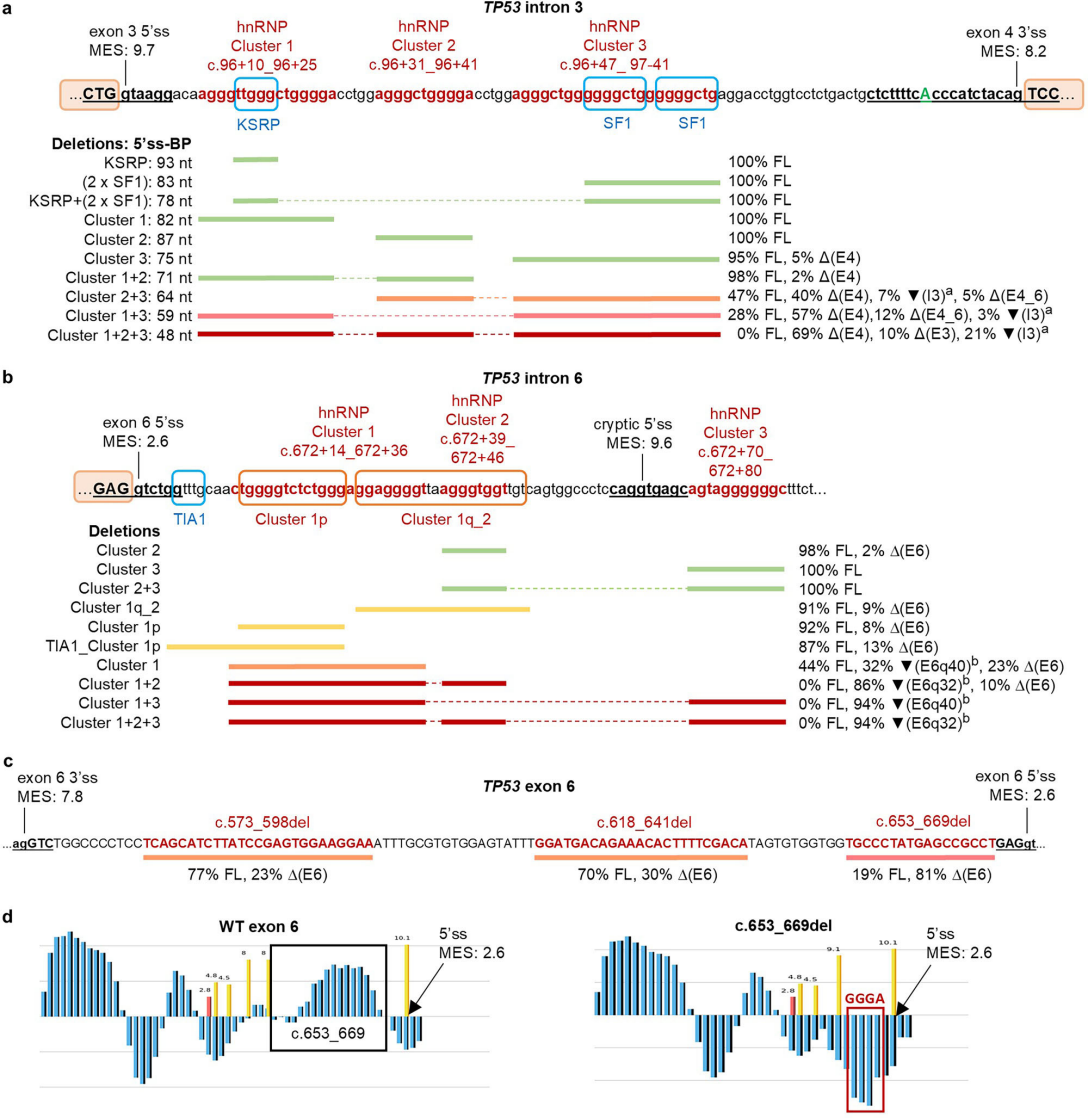
Microdeletion analysis of *TP53* intron 3, intron 6, and exon 6: SRE mapping and assay results. **a** Intron 3 G-rich intervals and predicted binding sites for splicing factors KSRP, SF1, and hnRNPs A1 or F/H group. The experimentally inferred canonical adenosine BP (c.97-12 A)^33^ is capitalized and in green font. ^a^The ▾(I3) transcripts retained the intron 3 sequence with intronic microdeletions. **b** Intron 6 predicted binding site for TIA1 splicing factor and G-rich intervals that are predicted binding sites for hnRNPs A1 or F/H group. ^b^The ▾(E6q32) and ▾(E6q40) transcripts were a product of intronic cryptic donor usage located at c.672+63 with MES score 9.6. The percentage of uncharacterized transcripts are not shown. **a**, **b** Solid lines represent the length and location of deletions, and broken lines linking the solid lines represent the combinations of deletions in a single construct. Green lines indicate no or negligible splicing impact. Deletions resulting in increasing levels of splicing aberration range from yellow (minimal impact), orange, pink, to red (complete impact). **c** ESE-rich intervals in exon 6 with different levels of splicing impact upon deletion. **d** HEXplorer profile of sequence upstream of exon 6 donor site (MES score 2.6); c.653_669del removes the ESE cluster in the WT sequence and creates an ESS motif (GGGA) that binds hnRNP F/H. See Supplementary Data 1 for detailed assay results.

HEXplorer analysis revealed several putative SRE-rich regions in intron 3, intron 6, and exon 6 (Supplementary Fig. S2). To determine if they play a role in splicing, we analyzed several intronic microdeletions spanning hnRNP clusters and putative KSRP, SF1, and TIA1 binding sites, and exon 6 microdeletions spanning putative ESE motifs (Fig. 2, Supplementary Data 1). Microdeletions affecting putative SREs were designed such that none were predicted to create a new donor or acceptor motif. Concerning introns 3 and 6, we focused on six clusters (three in each intron) of hnRNP A1 and H motifs predicted by SpliceAid in the G-rich regions (Fig. 2a, b). SpliceAid analysis of intron 3 also predicted the presence of binding sites for the splicing factors KSRP and SF1 that are involved in microexon recognition^23^. Moreover, SpliceAid predicted a binding site in intron 6 for the TIA1 protein that is known to promote the usage of weak donor sites^32^. We excluded exon 3 from the microdeletion analysis because of its small size of 22 nt.

#### Intron 3

The individual deletions of hnRNP cluster 1, hnRNP cluster 2, KSRP, and SF1 in intron 3, as well as the combined deletion of KSRP + SF1, had no effect on splicing (Fig. 2a). It had been previously reported that deletions of the two tracts of six Gs in intron 3 (c.96+53_97-52, c.97-49_97-44) induced intron 2 retention^12^, but we did not observe this event in our splicing assay of the hnRNP cluster 3 microdeletion spanning both of these G-runs (c.96+47_ 97-41del). Instead, c.96+47_ 97-41del induced exon 4 skipping (5% Δ(E4)).

The combined deletions of intron 3 hnRNP clusters also induced exon 4 skipping (2–69% Δ(E4)) and other minor aberrations including exon 3 skipping, intron 3 retention, and skipping of exons 4–6 (Fig. 2a). These deletions shortened intron 3, thus we assessed intron spatial constraint as an alternative mechanism for causing the splicing abnormalities. We measured the distance between the exon 3 donor and the experimentally inferred canonical adenosine BP in intron 3 located at c.97-12^33,34^. The consequent FL transcript depletion correlated with the donor-to-BP distance resulting from the deletions (Fig. 2a): clusters 1 + 2 (98% FL, 71-nt distance); 2 + 3 (47% FL, 64-nt distance); 1 + 3 (28% FL, 59-nt distance); and 1 + 2 + 3 (0% FL, 48-nt distance). Notably, the intron 3 cluster 1 + 2 + 3 deletion shortened the distance between the donor and the c.97-12 A BP from 97 nt to 48 nt, passing the previously established donor-to-BP length cutoff of <50 nt^35^ for critical risk of mis-splicing and profound splicing abnormalities.

To test the relevance of the intron 3 sequence in the splicing process, we replaced it with *ATM* intron 21 sequence, which has a similar size (114 nt) and does not contain G-runs. This chimeric construct only induced a low level of exon 4 skipping (1.3% Δ(E4), Supplementary Data 1), suggesting that splicing factors other than hnRNP F/H may be involved in *TP53* exon 3 and 4 recognition in the SKBR3 cell line. This intron 3 substitution assay did not result in intron 2 retention, also suggesting that G-quadruplex structures may not be necessary for normal splicing of this region. Moreover, the presence of a minor multi-exon skipping transcript lacking exons 4, 5 and 6 in the assay of intron 3 clusters 2 + 3 and 1 + 3 deletions (Fig. 2a) suggests some interdependence between these three exons for their recognition by the splicing machinery.

#### Intron 6

Analysis of intron 6 microdeletions revealed that the number of ISE clusters or G-runs downstream of the native donor influenced the level of weak donor site usage (Fig. 2b). Deletion of hnRNP cluster 2 only, and cluster 3 only, each containing one G-run, had negligible or no splicing impact (98–100% FL transcript). Deletion of intron 6 hnRNP cluster 1p (c.672+15_672+27del), and cluster 1q_2 (c.672+29_672+49del), each spanning two G-runs, led to minor exon 6 skipping (8–9% Δ(E6)). Extending the cluster 1p deletion to include the putative TIA1 binding site (c.672+7_672+27del) increased the Δ(E6) level to 13%. Deletion of cluster 1 (c.672+14_672+36del), spanning three G-runs, induced 23% Δ(E6) and activation of cryptic donor in intron 6 (32% ▾(E6q40)). Combined deletions of intron 6 hnRNP clusters 1 + 2, 1 + 3, and 1 + 2 + 3 did not produce any trace of the minigene FL transcript, suggesting that these G-rich sequences are critical for normal splicing of this region. Each of these combined deletions mainly activated the intronic c.672+63 cryptic donor (86–94% of overall expression). Moreover, cluster 1 + 2 deletion that effectively removed all four G-runs between the native and the cryptic donor resulted in complete inactivation of the native donor site. These results also suggest that the G-rich sequences have a greater contribution to exon 6 donor site recognition compared to the TIA1 binding site.

#### Exon 6

Microdeletion analysis of exon 6 HEXplorer-predicted ESE motifs (Fig. 2c) showed that the three selected microdeletions (c.573_598del, c.618_641del, and c.653_669del) affected exon 6 recognition (23–81% Δ(E6)), indicating ESE enrichment within these intervals. Additionally, c.653_669del (81% Δ(E6)) created a G-run upstream and adjacent to the weak native donor (Fig. 2d).

### Analysis of ClinVar-reported deletions

We selected and assayed deletions reported in ClinVar (Supplementary Data 2) that partially overlap with intron 3 hnRNP clusters 2 + 3 (c.96+31_96+54del) or located within the exon 6 ESE-enriched clusters (c.581_585del, c.628_639del and c.655_670del).

In our intron 3 microdeletion analysis (Fig. 2a), we showed that the hnRNP clusters 2 + 3 deletion altered splicing (47% FL), and we postulated that splicing alteration was not caused by deletion of specific hnRNP-targeted sequences, but rather by critical shortening between the exon 3 donor site and the canonical adenosine BP (c.97-12 A). The ClinVar-reported c.96+31_96+54del had minimal impact on splicing (95% FL). This deletion spanned intron 3 hnRNP clusters 2 and 3 (the latter, partially), shortening the distance between exon 3 donor and c.97-12 A to 73 nt, further supporting the claim that it was actually donor-to-BP distance that explains the splicing abnormality in the microdeletion experiments summarized in Fig. 2a. Of the three ClinVar-reported exon 6 deletions, only the c.655_670del variant had a major impact on splicing (Supplementary Fig. S3), mainly due to the creation of a new donor within exon 6 that produced an in-frame transcript with a 21 nt deletion (90% Δ(E6q21)).

### Analysis of single nucleotide variants

We assayed 134 SNVs, including four variants located at the splice donor/acceptor ±1,2 dinucleotide positions as positive controls, 66 SNVs in exon 3, 43 SNVs in exon 6, and 21 SNVs in intron 6 (Supplementary Data 2). Due to the exploratory nature of this study, we assayed all possible SNVs in exon 3 (from c.75 to c.96 totaling 66 variants) to check for exonic variants that affect the recognition of this microexon. We selected exon 6 SNVs with HEXplorer delta HZEI score ≤ –5 and located within the three microdeletions confirmed to result in exon skipping. For intron 6, we selected SNVs between the native and cryptic donors and outside the consensus motifs (c.672+7 to c.672+60) that passed at least two of the following filters: HEXplorer delta HZEI ≥ 5, PhyloP conservation score >0.5, or SpliceAI max delta score >0.1. Expectedly, 15 of the 21 intronic SNVs that passed these criteria are located within G-runs. We excluded exon 6 and intron 6 SNVs with gnomAD minor allele frequency >0.0001. SNVs that impact splicing ( < 95% FL transcript) are shown in Table 1.

**Table 1:** Percentage of transcripts produced by spliceogenic *TP53* SNVs

Variant	Exon (E)/Intron (I)	Full-length transcript	PTC transcripts	In-frame and other transcripts
c.75-2 A > G	I2	–	28% Δ(E3) 34% Δ(E3_E4p19)	31% Δ(E3_E4) 4% ▾(I2) 3% 785-nt^a^
c.82 G > T	E3	85%	5% Δ(E6) 10% Δ(E3)	–
c.96 G > T	E3	83%	17% Δ(E3)	–
c.96+1 G > T	I3	–	100% Δ(E3)	–
c.560-2 A > C	I5	–	78% Δ(E6p17) 17% Δ(E6)	3% ▾(I5) 3% 983-nt^a^
c.592 G > T	E6	74%	26% Δ(E6)	–
c.593 A > T	E6	80%	20% Δ(E6)	–
c.619 G > T	E6	91%	9% Δ(E6)	–
c.622 G > T	E6	84%	16% Δ(E6)	–
c.661 G > T	E6	92%	8% Δ(E6)	–
c.662 A > G	E6	92%	8% Δ(E6)	–
c.662 A > T	E6	90%	10% Δ(E6)	–
c.672+1 G > A	I6	-	90% ▾(E6q5) 6% Δ(E6)	2% 925-nt^a^, 2% 1005-nt^a^
c.672+7 T > C	I6	89%	11% Δ(E6)	–
c.672+7 T > G	I6	90%	10% Δ(E6)	–
c.672+18 G > A	I6	86%	14% Δ(E6)	–
c.672+26 G > C	I6	92%	8% Δ(E6)	–

The WT minigene mgTP53_2-9 produced 100% FL transcript. For variant minigene constructs, only transcripts accounting for >1% of the overall expression were included in the analysis. Percentages were rounded off to the nearest integer. See Supplementary Data 2 for detailed results of all assayed variants, and Supplementary Fig. S4 for fluorescent fragment analysis results for spliceogenic variants that produced <95% FL transcript.

*PTC* premature termination codon.

^a^Uncharacterized transcripts.

#### Control variants

We assayed four splice site dinucleotide variants flanking exon 3 (c.75-2 A > G, c.96+1 G > T) and exon 6 (c.560-2 A > C, c.672+1 G > A), assumed from position and bioinformatic prediction to impact splicing, as positive controls. The four variants totally disrupted splicing without any trace of the minigene FL transcript, generating at least 11 anomalous transcripts: Δ(E3), Δ(E3_E4p19), Δ(E3_E4), ▾(I2), Δ(E6p17), Δ(E6), ▾(I5) and ▾(E6q5), as well as three minor uncharacterized transcripts of 785, 925 and 1005 nucleotides. Only c.96+1 G > T induced complete exon skipping (100% Δ(E3)) while the remaining variants induced combinations of aberrant transcripts with alternative splice site usage, (multi-)exon skipping, or intron retention. The exon 6 acceptor site variant c.560-2 A > C principally generated Δ(E6p17) transcript (78%), resulting from the use of an exonic cryptic acceptor site 17 nt downstream strengthened by this variant (MES: 2.58 → 4.30). The exon 6 donor site variant c.672+1 G > A induced 90% ▾(E6q5) transcript due to the usage of a weak cryptic donor (MES 0.72) 5 nt downstream, whose recognition is likely promoted by the surrounding SREs.

#### Test variants

Of the exon 3 SNVs located outside of the acceptor and donor consensus motifs, only the c.82 G > T variant resulted in exon 3 skipping (10% Δ(E3)); this variant also had a minor effect on exon 6 recognition (5% Δ(E6)). The remaining exon 3 SNVs had no impact on splicing, except c.96 G > T (17% Δ(E3)), which is the last base of the exon and part of the donor consensus motif. Seven exon 6 SNVs (c.592 G > T, c.593 A > T, c.619 G > T, c.622 G > T, c.661 G > T, c.662 A > G, and c.662 A > T) and four intron 6 SNVs (c.672+7 T > C, c.672+7 T > G, c.672+18 G > A, and c.672+26 G > C) disrupted exon 6 recognition (8–26% Δ(E6)). The remaining exon 6 and intron 6 SNVs resulted in either very low level Δ(E6) transcript or 100% FL transcript.

#### DeepCLIP analysis

In order to identify the putative splicing factors involved in exon 3 and exon 6 recognition, and to determine the effect of SNVs on the binding of these factors, we ran DeepCLIP predictions for 12 SNVs outside of consensus splice site motifs that resulted in exon skipping (Table 2, Supplementary Data 3). The delta HZEI scores generally correlated with DeepCLIP predictions. For example, delta HZEI scores indicating enhancer motif loss had corresponding DeepCLIP-predicted decrease in the binding of enhancer proteins.

**Table 2:** HEXplorer delta HZEI and DeepCLIP analysis of SNVs outside the consensus splice site motifs and confirmed to result in exon skipping

Variant	Aberrant transcripts	Delta HZEI^a^	SR protein/hnRNP with decreased binding to ESE/ISE motif ^b^	SR protein/hnRNP that bind to created ESS/ISS motif ^b^	Other RBPs potentially involved in exon skipping^c^
c.82 G > T	10% Δ(E3); 5% Δ(E6)	−20.95	TRA2β (0.80)	–	U2AF2 (0.86,r)
c.592 G > T	26% Δ(E6)	−13.37	SRSF9 (0.97), TRA2α (0.90)	–	DAZAP1 (0.83,r)
c.593 A > T	20% Δ(E6)	−18.16	SRSF9 (0.98), SRSF10 (0.95), TRA2α (0.89)	–	DAZAP1 (0.84,r)
c.619 G > T	9% Δ(E6)	−17.05	–	hnRNP L (0.81)	–
c.622 G > T	16% Δ(E6)	−9.65	TRA2α (0.82)	–	DAZAP1 (0.89,r)
c.661 G > T	8% Δ(E6)	−12.12	–	hnRNP A1 (0.91)	PTBP1 (0.95,r), U2AF2 (0.82,r)
c.662 A > G	8% Δ(E6)	−6.92	–	–	U2AF2 (0.80,r)
c.662 A > T	10% Δ(E6)	−5.84	–	hnRNP L (0.83)	–
c.672+7 T > C	11% Δ(E6)	2.15	–	–	TIA1 (0.98,e) binding decreased by variant
c.672+7 T > G	10% Δ(E6)	−4.48	–	–	TIA1 (0.98,e) binding decreased by variant
c.672+18 G > A	14% Δ(E6)	18.31	hnRNP H2 (0.83)	–	–
c.672+26 G > C	8% Δ(E6)	14.81	hnRNP A1 (0.96), hnRNP H2 ( > 0.99)	SRSF7 (0.91)	–

^a^HEXplorer analysis of 11-nt sequences: negative delta HZEI score in the exon indicate ESE loss and/or ESS gain. Positive delta HZEI score in the intron indicate ISE loss and/or ISS gain.

^b^DeepCLIP-identified SR proteins and hnRNPs that regulate exon inclusion in a context-dependent manner; a splicing factor can either act as enhancer or repressor depending on the location of its binding site. The highest binding score for each RBP is enclosed in parenthesis.

^c^Other DeepCLIP-identified RBPs that may act as enhancers (e) or repressors (r). DAZAP1, PTBP1, and U2AF2 are predicted to bind to variant-created motifs. TIA1 is predicted to bind to the WT intronic sequence downstream of exon 6 donor site.

DeepCLIP analysis identified at least six SR proteins and hnRNPs that bind to SREs in WT *TP53* pre-mRNA (SRSF9, SRSF10, TRA2α, TRA2β, hnRNP A1, and hnRNP H2), ensuring normal splicing of exons 3 and 6. Six of the spliceogenic variants disrupted the ESE/ISE motifs targeted by these enhancer proteins. ESE disruption by exon 3 variant c.82 G > T decreased TRA2β binding resulting in decreased exon 3 inclusion. Similarly, exon 6 variants c.592 G > T, c.593 A > T, and c.622 G > T weakened the ESEs leading to decreased binding of SRSF9, SRSF10, or TRA2α. Intron 6 variants c.672+18 G > A and c.672+26 G > C disrupted the ISE motifs/G-runs resulting in decreased binding of hnRNPs A1 or H2. Predicted decreased binding of these enhancer proteins in exon 6 or intron 6 could explain the decreased exon 6 inclusion.

Five of the six variants that disrupted enhancer motifs (c.82 G > T, c.592 G > T, c.593 A > T, c.622 G > T, and c.672+26 G > C) also created binding sites for repressor proteins U2AF2, PTBP1, DAZAP1, or SRSF7. Ectopic binding of PPT splicing factors U2AF2 and PTBP1 in the exon can repress exon inclusion^36,37^. Binding of DAZAP1 to the exon^38^ and binding of SRSF7 downstream of the donor splice site^39^ can also inhibit exon inclusion. Moreover, we predicted four exonic variants (c.619 G > T, c.661 G > T, c.662 A > G and c.662 A > T) to alter splicing through ESS creation only, which then become target sites for U2AF2, PTBP1, hnRNP A1 or hnRNP L that would function as repressors in this context^36,37,40,41^.

In addition, we predicted the TIA1 protein to bind to the intronic sequence immediately downstream of the weak exon 6 donor site. TIA1 promotes the recognition of weak donor sites when bound to the downstream intronic uridine-rich sequences by stabilizing U1 snRNP recruitment^32^. The decrease in exon 6 inclusion resulting from c.672+7 T > C and c.672+7 T > G variants could be due to TIA1 binding site disruption. Notably, this putative TIA1 binding site is also a weak cryptic donor motif (MES 0.72), the one that generates the ▾(E6q5) transcript when activated. We hypothesize that TIA1 binding to this uridine-rich motif prevents the usage of this cryptic donor while simultaneously stabilizing U1 snRNP binding to the adjacent weak native donor site. In the event of native donor site abrogation, as in the case of c.672+1 G > A (Table 1), this nearby cryptic donor would then be used. We note that the TIA1 binding site alteration had a modest effect, from 8% when disrupted by SNVs (Table 2) to 13% when deleted (Fig. 2b), so it is likely that TIA1 is one more splicing factor of the set involved in splicing control of this region.

### Prediction of splicing impact: SpliceAI vs delta HZEI

We obtained SpliceAI predictions for all individual deletions and SNVs assayed (Supplementary Data 4, Fig. 3a). Expectedly, the four SNVs located at the donor/acceptor ±1,2 dinucleotide positions with complete splicing impact had the highest SpliceAI scores (0.98–1.0).

**Fig. 3:**
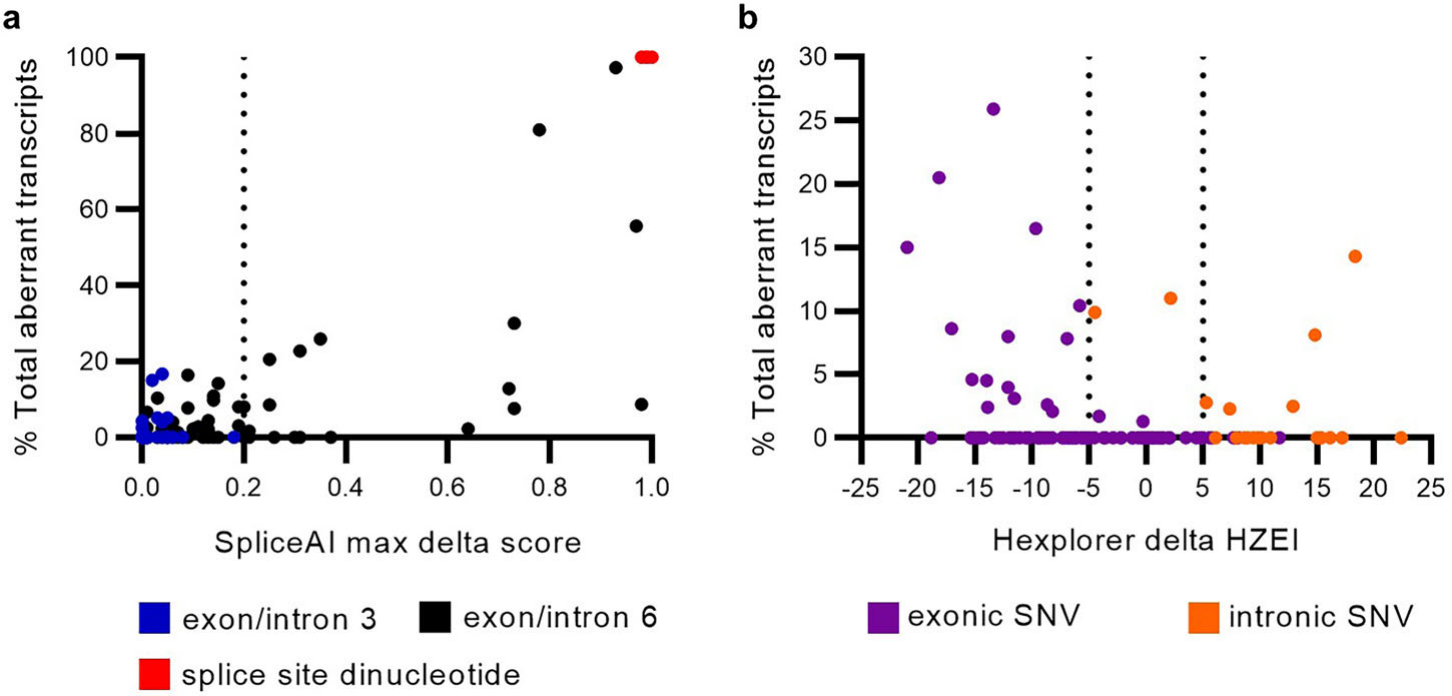
Splicing impact prediction. **a** SpliceAI scores and percentage of total aberrant transcripts for all individual deletions and SNVs assayed (*n* = 152). Variants with SpliceAI score ≥0.2 are predicted to alter splicing^30^. **b** HEXplorer delta HZEI scores and percentage of total aberrant transcripts for SNVs outside of splice consensus motifs (*n* = 112). Exonic SNVs with delta HZEI score ≤–5 and intronic SNVs with delta HZEI score ≥5 are predicted to disrupt SREs^62^.

We computed the likelihood ratio for spliceogenicity using the SpliceAI thresholds previously recommended for bioinformatic prediction of splicing impact for variants outside of the donor/acceptor ±1,2 dinucleotide positions, based on analysis of experimental data without quantification of transcript levels^30^. Likelihood ratio analysis based on quantitative data from this study, and selecting ≥5% aberrant transcript as a splice event (Table 3), generated similar findings to those reported previously^30^. The SpliceAI cutoff score of ≥0.2 equated to a moderate level of evidence for spliceogenicity. Of the 20 variants with SpliceAI score ≥0.2, 12 were true positive calls. Seven of these true positive calls, including five deletions (c.573_598del, c.618_641del, c.653_669del, c.655_670del, and c.672+14_672+36del) and two SNVs (c.592 G > T and c.593 A > T), led to >20% expression of aberrant transcripts. Regarding the mechanism of splicing impact of the 12 spliceogenic variants with SpliceAI score ≥0.2, seven were ESE/ISE motif deletions, four were SRE disruption by SNVs, and one was a deletion that created a new donor site. The remaining eight variants with SpliceAI score ≥0.2 were false positives and had aberrant transcript levels ranging from 0 to 2.3%. SpliceAI score >0.1 and <0.2 equated to uninformative strength of evidence, and the level of aberrant transcripts ranged from 0 to 14.3% for the 22 variants in this category. The SpliceAI cutoff score of ≤0.1 equated to a supporting level of evidence against spliceogenicity. It is notable that the eight variants in this category that were observed to impact splicing (i.e. false negative calls) had only 5.1–16.7% expression of aberrant transcripts. These eight false negatives were comprised of four SRE-disrupting SNVs, one deletion within an ESE cluster in exon 6, one exon 3 SNV within the donor consensus motif, and two intron 3 deletions that shortened the intron size. Interestingly, all exon 3 SNVs and intron 3 individual deletions had SpliceAI score <0.2, in general agreement with the assay results, suggesting a sequence context that ensures normal splicing or only minimal ( ≤ 16.7%) splicing aberration in this region.

**Table 3:** Likelihood ratio analysis of the maximum SpliceAI delta score for *TP53* deletions and SNVs outside the donor/acceptor ±1,2 dinucleotide positions using recommended thresholds

SpliceAI delta score	Splice event – no (<5% aberrant transcript)		Splice event – yes (≥5% aberrant transcript)		LR	Low CI	High CI	Evidence strength
	*n*	Proportion	*n*	Proportion				
≤0.1	98	0.79	8	0.33	0.42	0.24	0.75	Supporting evidence (non-spliceogenicity)
>0.1 and <0.2	18	0.15	4	0.17	1.15	0.43	3.09	Uninformative
≥0.2	8	0.06	12	0.50	7.75	3.55	16.92	Moderate evidence (spliceogenicity)

*CI* confidence interval, *LR* likelihood ratio, *n* number.

To compare the performance of bioinformatic tools in predicting spliceogenic SNVs that act through SRE-disruption, we selected 112 SNVs (12 spliceogenic and 100 non-spliceogenic) located outside of the consensus motifs (Supplementary Data 5). SpliceAI had a lower sensitivity (33%) than the HEXplorer 11-nt delta HZEI (83%) for spliceogenic SNVs that disrupt exonic or intronic SREs. However, SpliceAI had a higher specificity (76%) than the 11-nt delta HZEI (37%). While the delta HZEI score is useful in determining variants that cause ESE/ISE loss and/or ESS/ISS gain, this tool performed poorly at predicting SRE-disrupting variants that actually alter splicing. The delta HZEI cutoff score ≤ -5 for exon and ≥5 for intron had a high false positive rate of 63% for exonic and intronic variants outside of consensus splice motifs (Fig. 3b, Supplementary Data 5). For exonic SNVs only (8 spliceogenic and 83 non-spliceogenic), we observed that a different approach using delta HZEI cutoff score of ≤ –40 for whole exons would lower the false positive rate (35%) compared to the 11-nt delta HZEI (55%). The sensitivity of bioinformatic tools for splicing impact of exonic SRE-disrupting SNVs were: 100% for 11-nt delta HZEI, 88% for whole exon delta HZEI, and 38% for SpliceAI. Inversely, specificity for exonic SRE-disrupting SNVs were 80% for SpliceAI, 65% for whole exon delta HZEI, and 45% for 11-nt delta HZEI. Overall, SpliceAI and HEXplorer had limitations when predicting the splicing impact of variants acting via SRE mechanism.

## Discussion

We constructed the mgTP53_2-9 minigene containing *TP53* exons 2–9, a construct that can be used for splicing studies of any variant or motif within the inserted *TP53* region. This include variations that induce mRNA transcripts encoding truncated p53 isoforms, cause in-frame deletions, or generate transcripts sensitive to NMD. In this study, we used this minigene construct to examine the role of SREs on microexon 3 and exon 6 recognition. This is the first study that analyzed the effect of *TP53* deletions and SNVs on SRE motifs and the predicted binding of corresponding splicing factors. Our minigene assays that measured the splicing impact of deletions and SNVs within SRE-rich regions has provided mechanistic insights on normal and impaired splicing of the selected exons. Importantly, our splicing assay data can be used as evidence to support current assertions of variant pathogenicity or to re-classify variants of uncertain significance, as shown by three examples. First, the pathogenic assertion for c.655_670del in ClinVar was based on the assumption that it causes a translational frameshift. Here we showed that it actually affects function through aberrant splicing, which results in the removal of seven amino acids in a clinically relevant domain. Second, the likely benign classification for c.96+31_96+54del, originally curated based on a single submission with no assertion criteria provided, was supported by evidence of minimal splicing impact from this study. Third, the synonymous variant c.96 G > A (p.Leu32 = ) located at the donor consensus motif, currently classified as a variant of uncertain significance, can be downgraded to likely benign based on our assay result of no splicing impact. In addition, we verified that adequate donor-to-BP distance is required for normal splicing.

Exon 3, which has very strong acceptor (MES 11.65) and donor (MES 9.68) splice sites, was tolerant to disruption of SRE motifs within the exon and the downstream intron 3. Our minigene assays in SKBR3 cells showed that deletion of G-quadruplex structures in intron 3 did not induce intron 2 retention. Further, replacement of the entire intron 3 sequence with *ATM* intron 21 that is devoid of G-runs did not affect intron 2 excision in SKBR3 cells, supporting the notion that normal splicing of this region proceeds even in the absence of G-quadruplexes. These findings contrast previous results of intron 3 G-run deletion and mutagenesis experiments using a green fluorescent protein reporter containing *TP53* exons 2 to 4 assayed in H1299 cells^12^. This discordance in observations of intron 2 retention could possibly be ascribed to differences in cell lines and minigene construct design.

On the other hand, our findings showed the importance of intronic G-runs in exon 6 splicing, particularly in the activity of the weak exon 6 donor site (MES 2.6). There are five G-runs within the 85-nt intronic sequence directly downstream of exon 6. Deletion of three G-runs (hnRNP cluster 1) decreased the percentage of FL transcript by more than half; deletion of at least four G-runs (hnRNP clusters 1 + 2 or 1 + 3) was enough to completely inactivate the exon 6 donor site and activate the strong intronic cryptic donor site (MES 9.6). Meanwhile, creation of an exonic G-run directly upstream of the weak exon 6 donor site could explain the major splicing impact of c.653_669del, by acting as an ESS motif. Specifically, c.653_669del formed the GGGA motif that is the core binding site for hnRNP F/H^6^, potentially inhibiting the usage of the weak native donor. SpliceAid predicted the same splicing factors hnRNP F/H to target the intronic G-runs upstream of the c.672+63 cryptic donor, which may also play a role in inhibiting the usage of this cryptic site. Lastly, exon 6 ESE motif deletions resulted in decreased exon 6 inclusion, indicating that these ESE motifs also contributed to normal exon 6 splicing in addition to the intronic SREs.

Overall, we demonstrated that *TP53* deletions spanning multiple enhancer binding sites and located close to the regulated splice sites led to drastic splicing alterations. Whereas, SRE-disrupting SNVs induced weak splicing effects due to SRE motif redundancy, motif location, sequence context, or cooperation of several different splicing motifs and RBPs for exon recognition, similar to results of previous studies^42–44^. Therefore, in the context of germline variant classification, SRE-disrupting SNVs are less likely to confer pathogenicity via severe impact on mRNA processing. One intronic and 10 synonymous SNVs outside the splice consensus motif that were already reported in ClinVar did not alter splicing or had a minor splicing impact that was not clinically significant (FL transcript >85%); all were classified as likely benign (Supplementary Data 2). Of these, seven were predicted to alter SRE motifs. Nevertheless, there have been described SRE-disrupting SNVs that induce total splicing impact (no FL or negligible amounts), such as *CHEK2* c.883 G > A, c.883 G > T and c.884 A > T that were classified as pathogenic/likely pathogenic variants^45^.

SpliceAI, a deep neural network considered as one of the best splicing prediction tools to date^46^, positively detected all SRE-disrupting deletions and SNVs located outside of the splice consensus motifs that produced >20% aberrant transcripts, using the threshold of ≥0.2. This SpliceAI threshold was recommended conservatively for the application of supporting level of evidence for predicted impact on splicing within the ACMG/AMP framework^30^. Notably, likelihood ratio estimation derived from this dataset, enriched for SNVs and deletions affecting SRE motifs, provided further justification for SpliceAI score ≥0.2 as a conservative cutoff for the application of supporting level of bioinformatic evidence. SpliceAI that captures sequence features up to ±4999 nt surrounding a variant^47^, had a lower false positive rate than the SRE-specific tool HEXplorer delta HZEI, that only interrogates short stretches of nucleotide sequence. HEXplorer’s high false positive rate was probably due to SRE motif location and redundancy. Disruption of one enhancer motif while other enhancer motifs remain intact is less likely to cause a splicing aberration, especially if the motif is distantly located from the regulated splice site. However, SRE- and RBP-specific tools (HEXplorer, SpliceAid and DeepCLIP) are useful in mapping *cis*-regulatory elements and in unraveling the probable molecular mechanism and splicing factors underlying an experimentally observed splicing impact. Irrespective of the prediction tool selected, existence of both false positive and false negative predictions means that experimental assessment of variant impact on splicing remains important for variant classification.

We have identified the splicing factors TRA2β in exon 3; SRSF9, SRSF10, and TRA2α in exon 6; and hnRNP A1, hnRNP H2, and TIA1 in intron 6 as putative enhancers involved in *TP53* pre-mRNA splicing regulation. However, TRA2β may only have a minor role in exon 3 recognition as this exon is bounded by very strong acceptor and donor splice site motifs. We also identified putative repressor proteins that bind to variant-created silencer motifs including hnRNP A1, hnRNP L, SRSF7, DAZAP1, PTBP1, and U2AF2. It is possible that other splicing factors may be involved in regulating *TP53* splicing via SRE binding; we provide an extended list of DeepCLIP-identified splicing factors for the selected *TP53* regions in Supplementary Data 3.

We emphasize the role of predicted splicing factors targeting intron 6 elements to promote exon 6 donor site activity. Namely, TIA1 that binds directly downstream of the weak donor site, and hnRNP A1 and hnRNP H that bind to G-runs. TIA1, which is also a tumor suppressor^48^, has been implicated in p53 mRNA translational control in B cells by binding to the 3’ untranslated region^49^. A previous study showed that hnRNP F/H interacts with a G-quadruplex structure located at the *TP53* pre-mRNA polyadenylation signal in DNA-damaged cells; consequently, hnRNP F/H depletion compromises pre-mRNA 3′-end processing, p53 expression, and p53-mediated apoptosis^50^. Here, we predict that TIA1 and hnRNP H are also potentially involved in normal exon 6 splicing, preventing exon 6 skipping and/or intron 6 cryptic donor activation that would generate mRNA transcripts with a premature termination codon. It would be interesting to investigate the effect of depletion of TIA1, hnRNP A1, and hnRNP H, as well as the other splicing factors mentioned above, on *TP53* pre-mRNA splicing. It was previously suggested that deregulation of proper splicing factor balance in tumors, in addition to genetic variants affecting the *cis*-regulatory elements, could activate the intron 6 cryptic acceptor site leading to the expression of prometastatic p53Ψ isoform^14^. Future studies may also look into the potential production of a C-terminal truncated p53 isoform resulting from intron 6 cryptic donor activation upon splicing factor depletion (e.g. TIA1, hnRNP A1, and hnRNP H) or *cis*-regulatory element deletion/variation, and the functional role of such an isoform.

Outside of SRE studies, we explored the role of intron 3 spatial constraint in causing abnormal splicing. We confirmed findings from a previous minigene study of *HBB* intron 1^35^ that a donor-to-BP distance of <50 nt can cause completely abnormal splicing. This previous study also established a donor-to-BP distance cutoff of >60 nt for low risk of mis-splicing^35^. However, in our assay of *TP53* intron 3 cluster 2 + 3 deletion, we found that shortening the donor-to-BP distance to 64 nt still induced aberrant transcripts amounting to 53% of the overall expression. We also found that *TP53* intron 3 deletions with donor-to-BP distance of 71-75 nt resulted in low level exon 4 skipping (2–5%). That is, our findings indicated a longer donor-to-BP distance cutoff of >75 nt for low risk of abnormal splicing. Future studies using introns from multiple genes as models will further refine the donor-to-BP distance cutoffs for predicting risk for abnormal splicing.

It is worth noting that we used the SKBR3 breast cancer cell line in our assays. SKBR3 has a complex chromosome composition with widespread structural and numerical chromosomal abnormalities, including structural alterations affecting chromosome 17 where *TP53* is located^51^. We detected an abundant *TP53* transcript with r.401_1021del from our analysis of amplified RNA from SKBR3 (Supplementary Fig. S5) that could be explained by these genomic rearrangements. SKBR3 also harbors *TP53* p.Arg175His^52^, a gain-of-function variant classified as pathogenic in ClinVar. We demonstrated that testing of WT and four variant constructs in SKBR3 and two other cell lines with WT *TP53* status (HeLa^53^ and U2OS^54^) yielded identical splicing profiles (Supplementary Fig. S1), suggesting that the chromosomal alterations and the p.Arg175His variant inherently present in SKBR3 did not modify the splicing patterns of *TP53* mRNA produced by the minigene. That is, the background *TP53* mutation status of the SKBR3 cell line did not significantly impact spliceosome function in processing the minigene pre-mRNA. Moreover, the ▾(E6q5) transcript produced by the c.672+1 G > A control variant was also previously detected in the KG-1 human myeloid leukemia cell line that harbors this variant^55^. Nonetheless, we cannot exclude the possibility of varied splicing patterns produced by the minigene in other cell lines as different splicing factors may also be involved. For example, we did not detect the in-frame ▾(E6q18) transcript previously observed in the RT-PCR analysis of mRNA (without NMD inhibition) from peripheral blood mononuclear cells of a patient with Li–Fraumeni-like syndrome harboring the c.672+1 G > A germline variant^56^. This 18-nt partial intron retention was caused by the activation of another intronic cryptic donor that overlaps with the first G-run in intron 6 hnRNP cluster 1.

Our experiments focused on the characterization and semi-quantification of spliced transcripts generated by sequence changes in the *TP53* minigene construct. The construct included full intronic sequences, except those targeted by microdeletion analysis, to capture splicing elements across exons 2 to 9 to minimize the possibility of false results arising from incomplete splicing signals. Variants in exons 3 and 6 and their downstream introns would have adequate surrounding sequences to ensure reliable determination of variant-induced loss of native splice site activity for either exons or usage of cryptic splice site near or within these exons. To mitigate potential amplification bias favoring smaller cDNA fragments during transcript quantification, we lowered the number of PCR cycles down to the Ct value (26 cycles) determined by real-time PCR. Nonetheless, the minigene splicing assay may not accurately replicate the complexities of in vivo splicing due to its synthetic nature.

To enable application of splicing assay data for clinical variant classification, it is important to measure the proportions of normal and aberrant transcripts produced by the variants. This requires the use of cycloheximide as an NMD inhibitor in the assay to prevent the degradation of transcripts with a premature termination codon. The lack of NMD inhibitor would result in inaccurate measurements of spliced transcript levels and incorrect assignment of evidence weight in variant classification. We included appropriate assay controls to ensure that the measurement of variant-induced *TP53* transcripts were reliable. That is, we demonstrated that the WT construct control was spliced normally, while the positive control variants located at the splice site dinucleotide positions had complete splicing impact as expected. We also designed the assay to measure the mRNA transcribed from the minigene promoter only to assess splicing impact based on the canonical (MANE Select) transcript, the standard reference transcript for variant curation.

It was not appropriate to conduct a subsequent p53 protein analysis because the experimental design inherently blocked protein synthesis. Moreover, the minigene construct would produce a chimeric protein in the absence of a translational inhibitor. It was also not possible to measure potential dysregulation of naturally occurring Δ133 and Δ160 isoforms transcribed from the internal promoter, or the production of β and γ isoforms. These gaps offer opportunities for future research that will expand our understanding of the functional consequences of *TP53* variants that alter splicing. Such research may include experiments to determine whether the variants and deletions assayed in this study produce truncated p53 proteins, and if these truncated proteins influence *TP53* splicing. Another area for future investigation is assessing the potential modifying effect of common variants or polymorphisms on the splicing profile generated by the WT and variant alleles.

In conclusion, we provided splicing data for 27 deletions and 134 SNVs in the *TP53* gene. In addition, we incorporated intron substitution data as a control to verify our findings from microdeletion analysis that G-quadruplexes in intron 3 do not play a critical role in exon 3 recognition by the splicing machinery. We demonstrated that intron 6 G-runs hugely contribute to exon 6 splicing regulation. We also provided more data to inform prediction of splicing impact due to intronic deletions that shorten intron size. Therefore, intronic deletions outside the consensus splice motifs can have severe consequences and should be considered for splicing assays irrespective of bioinformatic prediction of impact especially if supported by clinical data. Our findings have potential implications for the identification of germline variants predisposing to hereditary cancer and somatic variants driving tumor development, and for elucidating the mechanisms of variant pathogenicity.

## Methods

### Bioinformatics and databases

Variant data and alternative transcripts were annotated based on the *TP53* MANE Select transcript (NM_000546.6), composed of 11 exons that encodes the canonical p53α protein. Splicing events were described with a short descriptor combining the following symbols: ∆ (skipping of exonic sequences), ▾ (inclusion of intronic sequences), E (exon), p (acceptor site shift) and q (donor site shift)^57,58^. When necessary, the number of deleted or inserted nucleotides is indicated. For example, ▾(E6q5) indicates the use of an intronic cryptic donor 5 nt downstream of exon 6, generating a 5-nt insertion into the mature mRNA.

*TP53* sequences spanning exon 3, intron 3, exon 6, and part of intron 6 (from c.672+1 to c.672+85) were analyzed with the following online in silico prediction tools for SREs: (i) HEXplorer for mapping out the putative enhancer and silencer regions^59^; (ii) SpliceAid, a database of experimentally derived target RNA sequences of RBPs^60^; and (iii) DeepCLIP, a deep learning approach, which integrates RNA binding functional data to predict the presence of splicing factor motifs^61^. In addition to SRE mapping, these tools were used to predict the impact of deletions and SNVs on putative SRE motifs and on RBP binding. For HEXplorer analysis of SNVs, the delta HZEI score of 11-nt sequences containing the SNV in the middle was calculated; delta HZEI ≤ -5 (exonic) or ≥5 (intronic) was considered as SRE-disrupting^62^. A similar approach of analyzing 11-nt sequences was used to generate DeepCLIP predictions for SNVs. Cutoffs for DeepCLIP analysis were set as follows: RBP binding score >0.8 for putative RBP binding site in the wild type and variant sequences, and a decrease in binding score by 0.3 or greater for binding site disruption. RBPs considered as splicing factors that regulate splice site usage by binding to exonic or intronic motifs, and also listed in SpliceAid were included. RBPs that bind to both wild type and variant sequences were excluded.

Splicing predictions were done using SpliceAI v1.3.1^47^ with maximum distance of ±4999 nt flanking the variant, unmasked, and GRCh38 genome assembly. SpliceAI cutoff scores were ≥0.2 for variants predicted to be spliceogenic (i.e. to impact native splicing patterns) and ≤0.1 for variants predicted to be non-spliceogenic^30^. The strength of the acceptor and donor consensus motifs was predicted by MaxEntScan (MES)^63^. PhyloP^64^ was used to measure nucleotide conservation of intronic SNVs. Minor allele frequency of SNVs was obtained from the gnomAD v2.1.1 non-cancer dataset^65^. Clinical variants and their pathogenicity assertions (as of 19 March 2025) were obtained from the ClinVar database^66^. ClinVar accession numbers were listed in Supplementary Data 2.

### Minigene construct design and site-directed mutagenesis

A 3487-bp insert, including exons 2–9, was designed in-house (Supplementary Fig. S6) and then generated by gene synthesis (GeneArt, Thermo Fisher Scientific, Waltham, MA, USA). This fragment was cloned into the pSAD splicing vector between the restriction sites *Sac*I and *Eco*RI to obtain the minigene mgTP53_2-9 (Fig. 1a), which was confirmed by sequencing (Macrogen, Madrid, Spain).

Microdeletions and candidate variants (Supplementary Data 6) were introduced into the wild type (WT) construct by site-directed mutagenesis using the QuikChange Lightning Kit (Agilent, Santa Clara, CA, USA). Mutant minigenes were confirmed by sequencing (Macrogen). Additionally, the G-rich *TP53* intron 3 was replaced with *ATM* intron 21 (as a control/reference intron which does not contain G-runs) (Supplementary Fig. S7) by overlap extension PCR as previously described^67^.

### Splicing assays

#### Transfection and RNA isolation

Approximately 2 × 10^5^ cells of the human breast cancer cell line SKBR3 (ATCC HTB-30) were grown in 0.5 mL of medium (Minimum Essential Medium -MEM-, 10% Fetal Bovine Serum, 1% nonessential amino acids, 2 mM Glutamine and 1% Penicillin/Streptomycin; Sigma-Aldrich, St. Louis, MO, USA) in 4-well plates (Nunc, Roskilde, Denmark). SKBR3 cells were transiently transfected with 1 µg of the WT/mutant minigenes and 2 µL of lipofectamine LTX (Life Technologies, Carlsbad, CA, USA).

In order to inhibit nonsense-mediated decay (NMD), a 4-h incubation with 300 µg/ml of cycloheximide (Sigma-Aldrich, St. Louis, MO, USA) was carried out 48 h after transfection. RNA Purification was performed using the Genematrix Universal RNA Purification Kit (EURx, Gdansk, Poland), with on-column DNAse I digestion.

To check splicing profile reproducibility, the WT and four variant constructs (c.76 C > A, c.592 G > T, c.655_670del, and [c.672+14_672+36del;c.672+39_672+46del]) were also tested in U2OS osteosarcoma and HeLa cell lines following the same protocols as above.

#### Reverse transcription polymerase chain reaction (RT-PCR)

A total of 400 ng of RNA were retrotranscribed with the RevertAid First Strand cDNA Synthesis Kit (Life Technologies), using the vector-specific primer RT-PSPL3-RV (5ʹ-TGAGGAGTGAATTGGTCGAA-3ʹ) and the manufacturer’s protocol.

The resulting cDNA was amplified with primers SD6-PSPL3_RT-FW (5ʹ-TCACCTGGACAACCTCAAAG-3ʹ) and RTpSAD-RV (CSIC Patent P201231427) using Platinum Taq DNA polymerase (Life Technologies) and the following cycling conditions: 94 °C/2 min, 35 cycles x [94 °C/30 s, 60 °C/30 s, 72 °C/(1 min/kb)], 1 cycle x [72 °C/5 min]. RT-PCR products were sequenced by Macrogen. The expected size of the minigene full-length (FL) transcript was 1202-nt.

In order to quantify the relative proportions of each transcript, semi-quantitative fluorescent RT-PCRs (26 cycles) were carried out in triplicate using Platinum Taq DNA polymerase (Life Technologies) and the primers SD6-PSPL3_RT-FW and RTpSAD-RV (FAM-labeled). Fluorescent products were run with LIZ-1200 size standard at the Macrogen facility and analyzed using Peak Scanner_V1.0 (Life Technologies). Only peak heights ≥100 RFU (relative fluorescence units) were considered. Mean peak areas of each transcript and standard deviations were calculated. The identity of the most abundant transcripts generated by microdeletions and variants was confirmed by Sanger sequencing.

### URL of databases and online tools

ClinVar (https://www.ncbi.nlm.nih.gov/clinvar/)

DeepCLIP (https://deepclip-web.compbio.sdu.dk/)

HEXplorer (https://rna.hhu.de/HEXplorer/)

gnomAD (https://gnomad.broadinstitute.org/)

Likelihood ratio for spliceogenicity (https://gwiggins.shinyapps.io/lr_shiny/)

MaxEntScan (http://hollywood.mit.edu/burgelab/maxent/Xmaxentscan_scoreseq.html)

SpliceAid (http://www.introni.it/splicing.html).

## Supplementary information

Supplementary Information

Supplementary Data

## Data Availability

All data generated or analyzed during this study are included in this published article and its supplementary files. All Sanger sequencing files (for minigene constructs and cDNAs from spliced transcripts generated by microdeletions and variants) and fragment analysis data can be accessed at Digital CSIC (https://digital.csic.es/handle/10261/364196 and 10.20350/digitalCSIC/16498).
