# Supplementary material for: TP53 minigene analysis of 161 sequence changes provides evidence for role of spatial constraint and regulatory elements on variant-induced splicing impact
Source: NPJ Genom Med. 2025 May 8;10:37. doi: 10.1038/s41525-025-00498-0 (PMC12062376; doi:10.1038/s41525-025-00498-0)
Supplement: Supplementary file 1 — Supplementary Information [file 41525_2025_498_MOESM1_ESM.pdf]

Supplementary Data Titles

Note: The contents of the following Supplementary Data Tables are provided in a separate Excel file.

Supplementary Data 1. Functional mapping of splicing regulatory elements in *TP53* intron 3, intron 6 and exon 6 by microdeletion assays.

Supplementary Data 2. Minigene assay results for *TP53* single nucleotide variants and ClinVar-reported deletions.

Supplementary Data 3. DeepCLIP analysis of 12 SNVs outside the consensus splice site motifs and confirmed to result in ≥5% exon skipping.

Supplementary Data 4. Percentage of aberrant transcripts induced by variants and their corresponding HEXplorer delta HZEI and SpliceAI max delta scores.

Supplementary Data 5. Performance evaluation of HEXplorer delta HZEI and SpliceAI max delta scores.

Supplementary Data 6. Microdeletions and mutagenesis primers.

SUPPLEMENTARY FIGURES

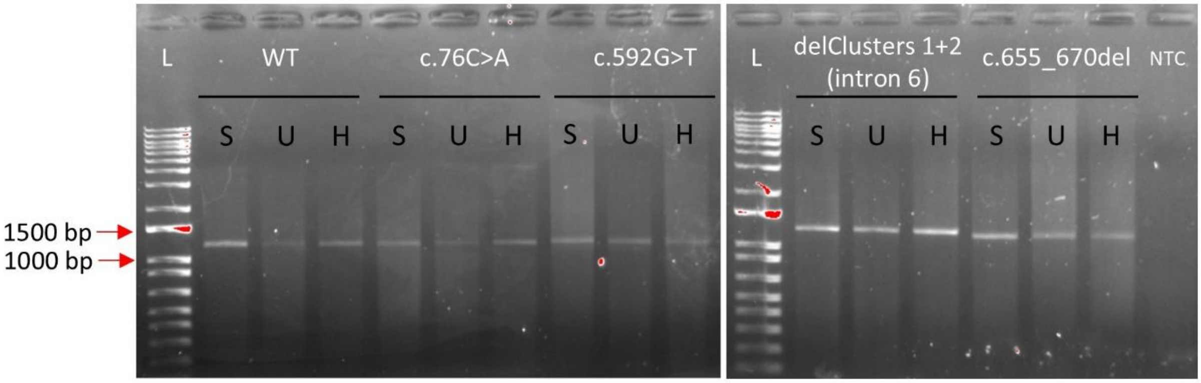

Supplementary Figure S1. Splicing profile reproducibility in SKBR3, U2OS, and HeLa cell lines. Agarose gel image showing splicing profiles generated by WT minigene mgTP53_2-9 and four variant constructs: c.76C>A, c.592G>T, c.655_670del, and intron 6 delClusters 1+2 [c.672+14_672+36del;c.672+39_672+46del]. L, Ladder; NTC, no template control; S, SKBR3; U, U2OS; H, HeLa.

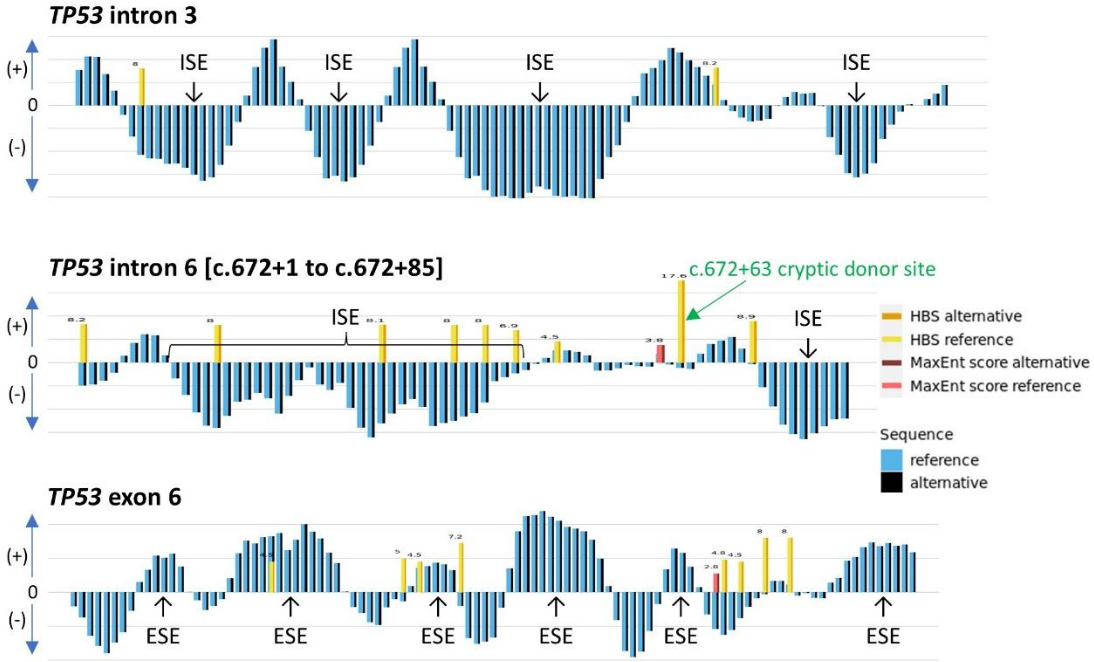

Supplementary Figure S2. **HEXplorer profile of *TP53* intron 3, intron 6, and exon 6 WT sequence.** For introns 3 and 6, clusters of negative HEXplorer scores (black/blue bars below zero) represent putative intronic splicing enhancers (ISEs). For exon 6, clusters of positive HEXplorer scores (black/blue bars above zero) represent putative exonic splicing enhancers (ESEs). Cryptic donor (orange/yellow) and acceptor (brown/pink) sites are also shown. None of the cryptic splice sites are predicted as strong motifs except for the c.672+63 cryptic donor site in intron 6.

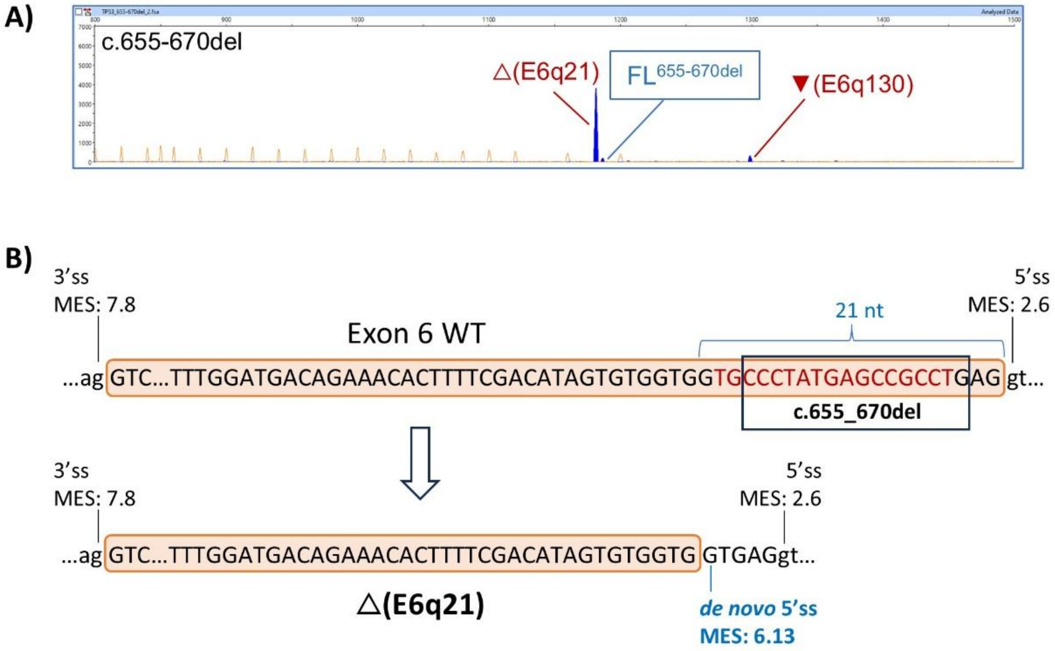

Supplementary Figure S3. **Minigene splicing assay result for the ClinVar-reported deletion c.655_670del. A)** Fluorescent fragment analysis result. FAM-labelled products (transcripts, blue peaks; $FL^{655\text{-}670del}$, minigene full-length transcript with the deletion) were run with LIZ1200 (orange peaks) as size standard. The x-axis indicates size in bp and the y-axis represents Relative Fluorescence Units (RFU). **B)** Schematic representation of *de novo* donor site creation within exon 6 owing to c.655_670del (boxed). Usage of the new donor site located 21 nt upstream of the weak exon 6 donor site produced the △(E6q21) in-frame transcript. The ESE-rich sequence affected by c.655_670del is shown in red font.

A)

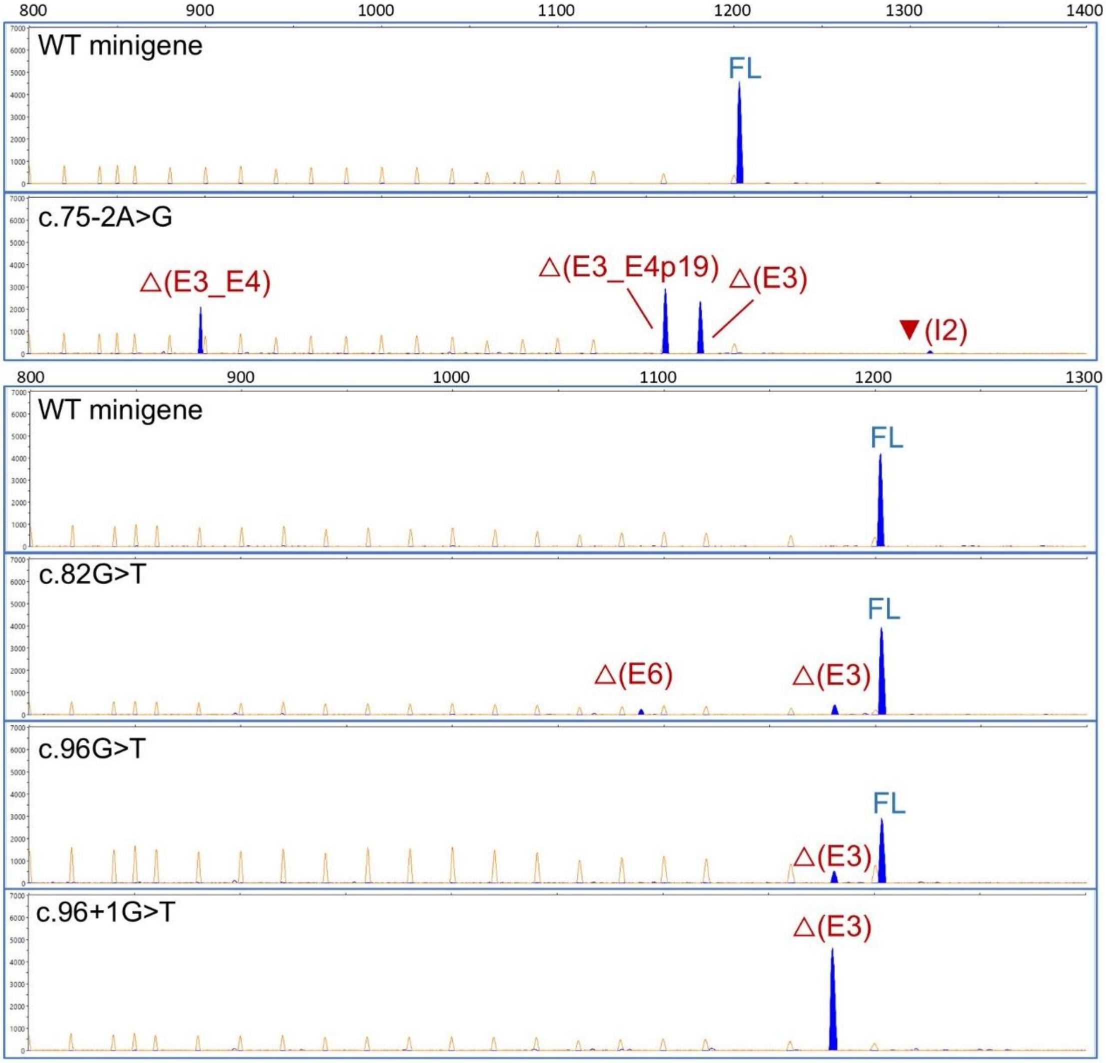

Supplementary Figure S4. Fluorescent fragment analysis results of spliceogenic single nucleotide variants. Results for variants that produced <95% full-length transcript and located in: **A)** Exon 3 and flanking splice site ±1,2 dinucleotide positions; **B)** Exon 6 and flanking splice site ±1,2 dinucleotide positions; **C)** Intron 6. FAM-labelled products (transcripts, blue peaks; FL, minigene full-length transcript) were run with LIZ1200 (orange peaks) as size standard. The x-axis indicates size in bp and the y-axis represents Relative Fluorescence Units (RFU).

Note: Supplementary Figure S4 panels B and C are in the next pages.

B)

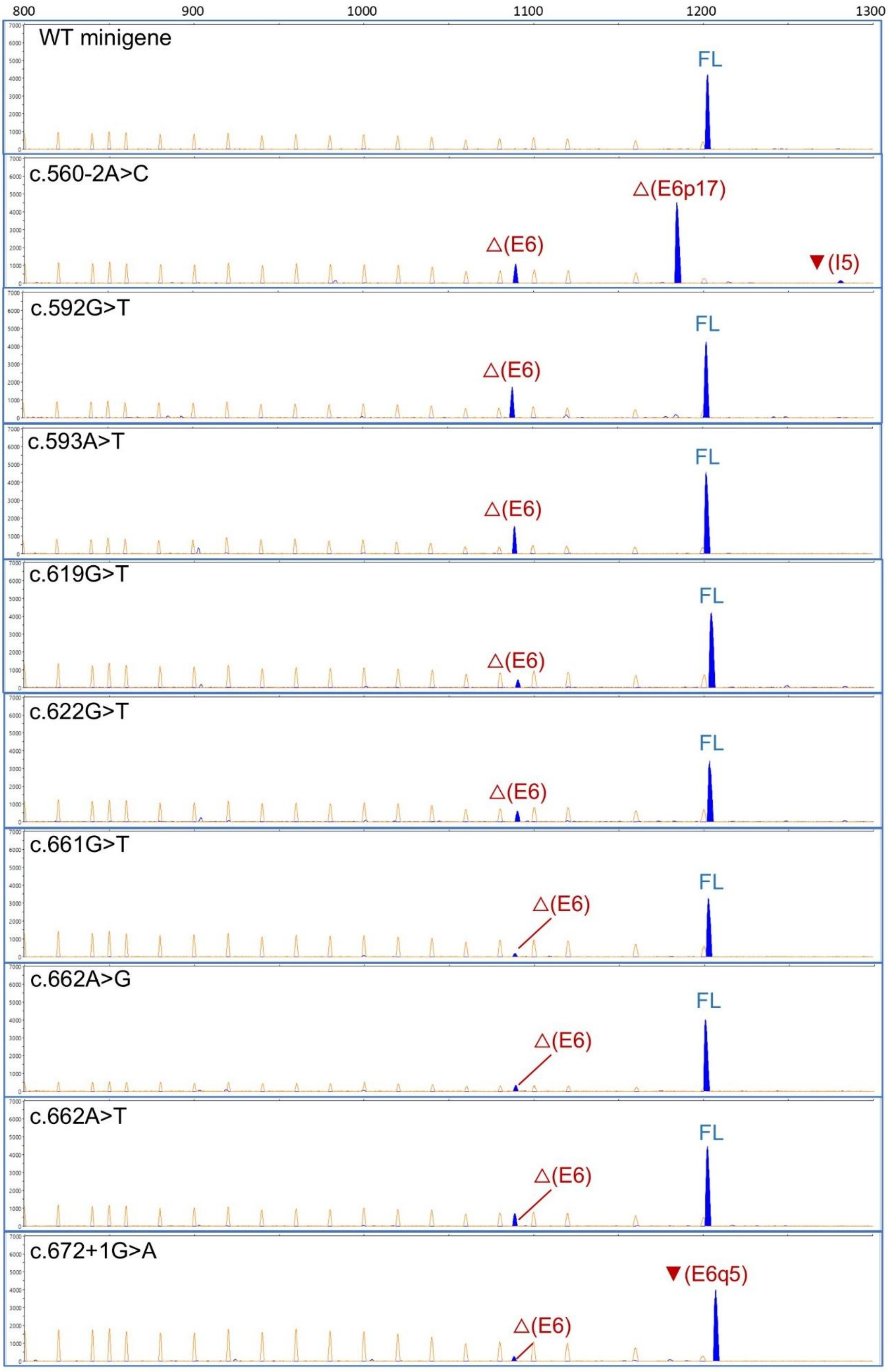
800
900
1000
1100
1200
1300
WT minigene
FL
c.560-2A>C
△(E6p17)
△(E6)
▼(I5)
c.592G>T
FL
△(E6)
c.593A>T
FL
△(E6)
c.619G>T
FL
△(E6)
c.622G>T
FL
△(E6)
c.661G>T
FL
△(E6)
c.662A>G
FL
△(E6)
c.662A>T
FL
△(E6)
c.672+1G>A
▼(E6q5)
△(E6)

C)

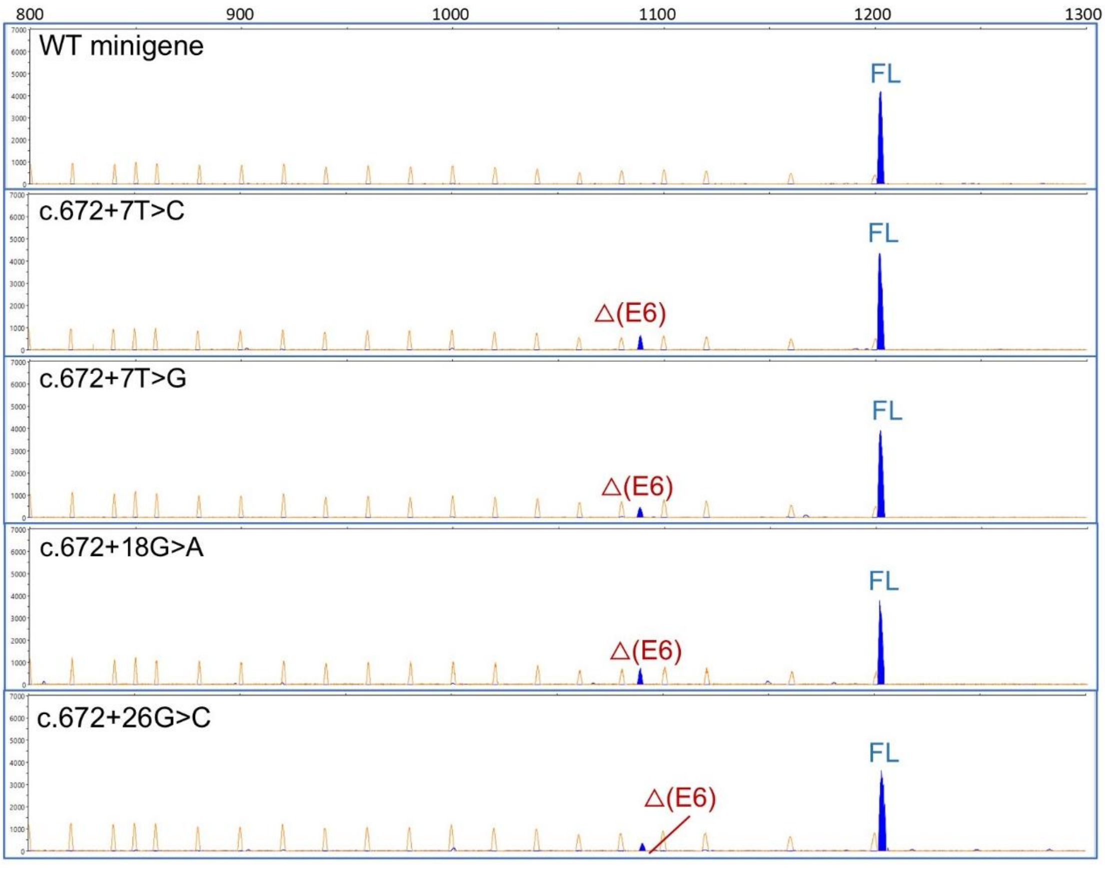
800
900
1000
1100
1200
1300
WT minigene
FL
c.672+7T>C
FL
△(E6)
c.672+7T>G
FL
△(E6)
c.672+18G>A
FL
△(E6)
c.672+26G>C
FL
△(E6)

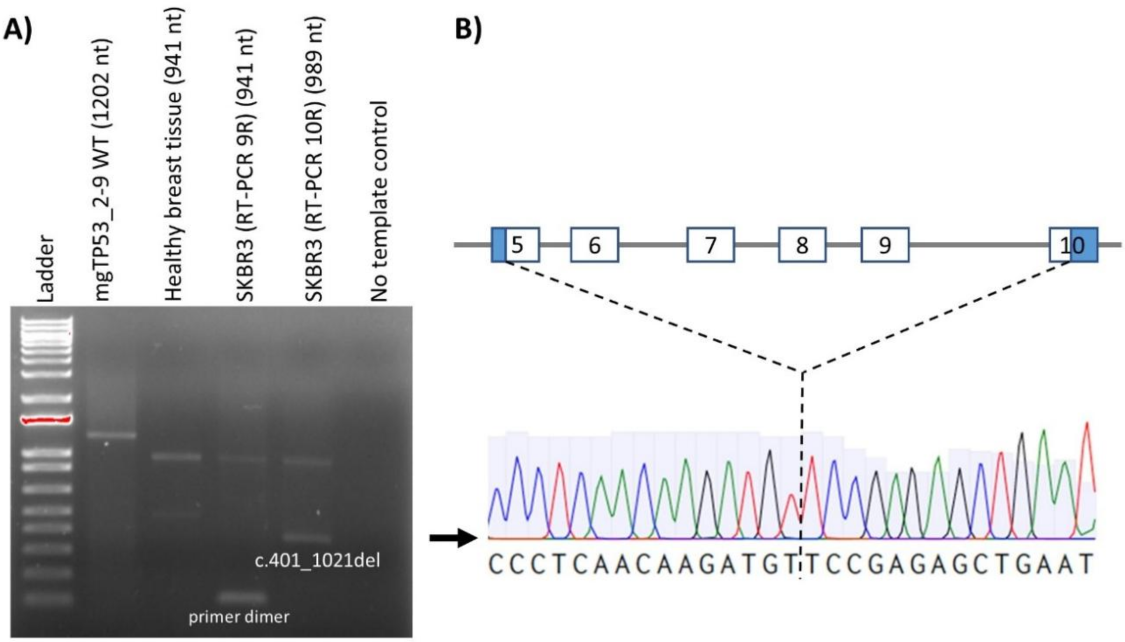

Supplementary Figure S5. **RT-PCR and Sanger sequencing of *TP53* mRNA from SKBR3 cells. A)** Agarose gel image showing amplified cDNAs from the mgTP53_2-9 minigene, healthy breast tissue, and SKBR3 cells. *TP53* mRNA from healthy breast tissue and SKBR3 cells was retrotranscribed using the primer RT_TP53_ex9R (5'-GGTGAAATATTCTCCATCCA-3'). The resulting cDNA from healthy breast tissue and SKBR3 cells was then amplified with primers RT_TP53_ex2F (5'-AGGAAACATTTTCAGACCTA-3') and RT_TP53_ex9R. For the *TP53* mRNA from SKBR3 cells, an additional retrotranscription experiment was done using the primer RT_TP53_ex10R (5'-ATTCAGCTCTCGGAACATCT-3'), and the cDNA was amplified with primers RT_TP53_ex2F and RT_TP53_ex10R. The RT-PCR of SKBR3 *TP53* mRNA retrotranscribed from exon 10 showed a shorter fragment. **B)** Sanger sequencing of the shorter fragment from SKBR3 cells revealed a deletion (c.401_1021del) that removes all of exons 6 to 9 and portions of exons 5 and 10. The numbered boxes represent exons that were partially or fully deleted (no shade). Broken lines indicate the precise locations of the deletion breakpoints.

SacI
GAGCTCcttgggttgtggtgaaacattggaagagagaatgtgaagcagccattcttttcctgctccacaggaagccgagctgtct
cagacactggcatggtgttggggagggggttccttctctgcaggcccaggtgacccagggttggaagtgtctcatgctggatccc
cacttttcctcttgcagCAGCCAGACTGCCTTCCGGGTCACTGCCATGGAGGAGCCGCAGTCAGATCCT Ex2
AGCGTCGAGCCCCCTCTGAGTCAGGAAACATTTTCAGACCTATGGAAACTgtgagtggatccattggaa
gggcaggcccaccacccccaccccaaccccagccccctagcagagacctgtgggaagcgaaaattccatgggactgactttctgc
tcttgtctttcagACTTCCTGAAAACAACGTTCTGgtaaggacaagggttgggctggggacctggagggctggggacct Ex3
ggagggctgggggctgggggggctgaggacctggtcctctgactgctcttttcacccatctacagTCCCCCTTGCCGTCCCA
AGCAATGGATGATTTGATGCTGTCCCCGGACGATATTGAACAATGGTTCACTGAAGACCCAGGT
CCAGATGAAGCTCCCAGAATGCCAGAGGCTGCTCCCCCCGTGGCCCCTGCACCAGCAGCTCCTAC Ex4
ACCGGCGGCCCCTGCACCAGCCCCCTCCTGGCCCCTGTCATCTTCTGTCCCTTCCCAGAAAACCTA
CCAGGGCAGCTACGGTTTCCGTCTGGGCTTCTTGCATTCTGGGACAGCCAAGTCTGTGACTTGCA
CGgtcagttgccctgaggggctggcttccatgagacttcaatgcctggccgtatccccctgcatttcttttgtttggaactttgggatt
cctcttcacccttggcttcctgtcagtgtttttttatagtttacccacttaatgtgtgatctctgactcctgtcccaaagttgaatattcc
ccccttgaatttgggcttttatccatcccatcacaccctcagcatctctcctggggatgcagaacttttctttttcttcatccacgtgtat
tccttggcttttgaaaataagctcctgaccaggcttggtggctcacacctgcaatcccagcactctcaaagaggccaaggcaggca
gatcacctgagcccaggagttcaagaccagcctgggtaacatgatgaaacctcgtctctacaaaaaaatacaaaaaattagcca
ggcatggtggtgcacacctatagtcccagccacttaggaggctgaggtgggaagatcacttgaggccaggagatggaggctgca
gtgagctgtgatcacaccactgtgctccagcctgagtgacagagcaagaccctatctcaaaaaaaaaaaaaaaaaagaaaagct
cctgaggtgtagacgccaactctctctagctcgctagtgggttgcaggaggtgcttacgcatgtttgtttctttgctgccgtcttccag
ttgctttatctgttcacttgtgccctgactttcaactctgtctccttcctcttcctacagTACTCCCCTGCCCTCAACAAGAT
GTTTTGCCAACTGGCCAAGACCTGCCCTGTGCAGCTGTGGGTTGATTCCACACCCCCGCCCGGCA Ex5
CCCGCGTCCGCGCCATGGCCATCTACAAGCAGTCACAGCACATGACGGAGGTTGTGAGGCGCTG
CCCCCACCATGAGCGCTGCTCAGATAGCGATGgtgagcagctggggctggagagacgacagggctggttgccca
gggtccccaggcctctgattcctcactgattgctcttagGTCTGGCCCCTCCTCAGCATCTTATCCGAGTGGAAGG
AAATTTGCGTGTGGAGTATTTGGATGACAGAAACACTTTTCGACATAGTGTGGTGGTGCCCTAT Ex6
GAGCCGCCTGAGgtctggtttgcaactggggtctctgggaggaggggttaagggtggttgtcagtggccctccaggtgagca
gtaggggggctttctcctgctgcttatttgacctccctataaccccatgagatgtgcaaagtaaatgggtttaactattgcacagttg
aaaaaactgaagcttacagaggctaagggcctcccctgcttggctgggcgcagtggctcatgcctgtaatcccagcactttgggag
gccaaggcaggcggatcacgaggttgggagatcgagaccatcctggctaacggtgaaaccccgtctctactgaaaaatacaaaa
aaaaattagccgggcgtggtgctgggcacctgtagtcccagctactcgggaggctgaggaaggagaatggcgtgaacctgggcg
gtggagcttgcagtgagctgagatcacgccactgcactccagcctgggcgacagagcgagattccatctcaaaaaaaaaaaaaa
aaggcctcccctgcttgccacaggtctccccaaggcgcactggcctcatcttgggcctgtgttatctcctagGTTGGCTCTGAC
TGTACCACCATCCACTACAACTACATGTGTAACAGTTCCTGCATGGGCGGCATGAACCGGAGGC Ex7
CCATCCTCACCATCATCACACTGGAAGACTCCAGgtcaggagccacttgccaccctgcacactggcctgctgtgcc
ccagcctctgcttgcctctgacccctgggcccacctcttaccgatttcttccatactactacccatccacctctcatcacatccccggc
ggggaatctccttactgctcccactcagttttcttttctctggctttgggacctcttaacctgtggcttctcctccacctacctggagctg
gagcttaggctccagaaaggacaagggtggttgggagtagatggagcctggtttttaaatgggacaggtaggacctgatttcctt
actgcctcttgcttctcttttcctatcctgagtagTGGTAATCTACTGGGACGGAACAGCTTTGAGGTGCGTGTT
TGTGCCTGTCCTGGGAGAGACCGGCGCACAGAGGAAGAGAATCTCCGCAAGAAAGGGGAGCCT Ex8
CACCACGAGCTGCCCCCAGGGAGCACTAAGCGAGgtaagcaagcaggacaagaagcggtggaggagaccaa
gggtgcagttatgcctcagattcacttttatcacctttccttgcctctttcctagCACTGCCCAACAACACCAGCTCCTCT
CCCCAGCCAAAGAAGAAACCACTGGATGGAGAATATTTCACCCTTCAGgtactaagtcttgggacctctta Ex9
tcaagtggaaagtttccagtctaacactcaaaatgccgttttcttcttgactgttttacctgcaattggggcatttgccatcaggggc
agtgatgcctcaaagacaatggctcctggttgtagctaactaacttcagaacaccaacttataccataatatatattttaaaggacc
agaccagctttcaaaaagGAATTC
EcoRI

Supplementary Figure S6. **Insert sequence of minigene mgTP53_2-9.** Exons 2 to 9 are shown in orange and upper case, cloning sites (SacI/EcoR1) are indicated in green and underlined and *Alu* sequences are yellow-shadowed. Size of the insert: 3487 bp. Structure: SacI - ivs1 (182 bp) – ex2 (102 bp) – ivs2 (117 bp) – ex3 (22 bp) – ivs3 (109 bp) – ex4 (279 bp) – ivs4 (757 bp) – ex5 (184 bp) – ivs5 (81 bp) – ex6 (113 bp) – ivs6 (568 bp) – ex7 (110 bp) – ivs7 (343 bp) – ex8 (137 bp) – ivs8 (92 bp) – ex9 (74 bp) – ivs9 (217 bp) – EcoR1.

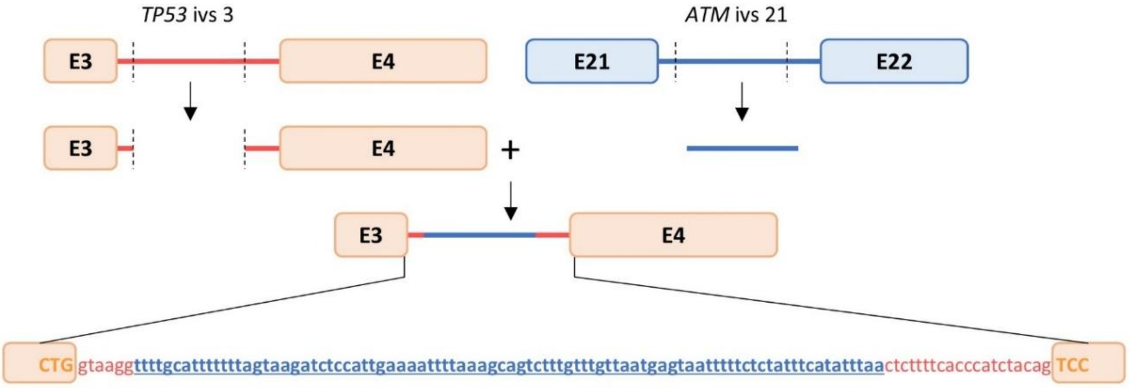

Supplementary Figure S7. **Replacement of *TP53* intron 3 with *ATM* intron 21.** Schematic representation of substitution of *TP53* intron 3 by *ATM* intron 21 (shown in blue), maintaining the canonical splice sites of *TP53* exons 3 and 4. The rest of the insert sequence of the mgTP53_2-9 is the same as the one shown in the Supplementary Figure S6.

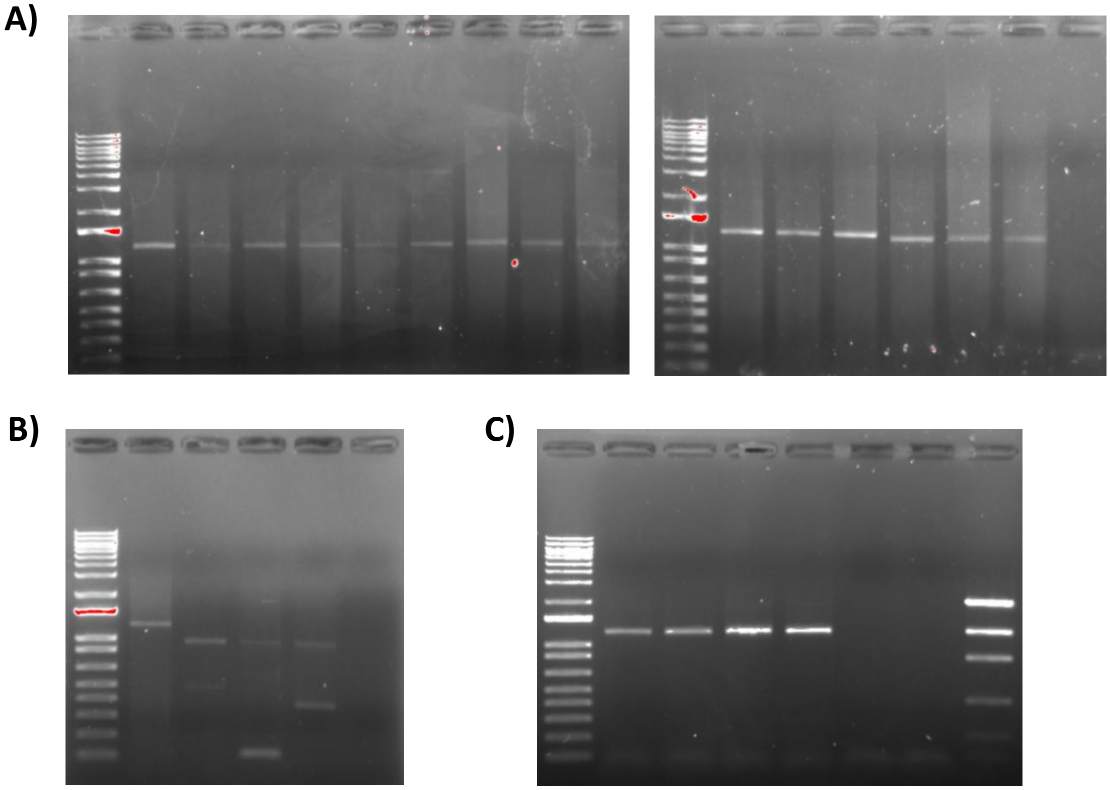

Supplementary Figure S8. **Uncropped and unprocessed agarose gel images.** These gel images were incorporated in: **A)** Supplementary Figure S1, **B)** Supplementary Figure S5, and **C)** Figure 1. The gel image in Figure 1 was cropped to include only lane 1 (ladder) and lane 2 (mgTP53_2-9 WT); samples in lanes 3-8 of the uncropped image C were as follows: 3 - c.84A>T, 4 - deletion of the KSRP binding site, 5 - deletion of the SF1 binding site (x2), 6 - negative control for RT-PCR, 7 - negative control for amplification, and 8 - low DNA mass ladder.
